# Tracing an Unyielding Work Compulsion: A Moderated Mediation Model of Abusive Supervision and Compulsory Citizenship Behavior

**DOI:** 10.3389/fpsyg.2021.746823

**Published:** 2021-11-29

**Authors:** Ali T. Baig, Zahid Riaz

**Affiliations:** Lahore School of Economics, Lahore, Pakistan

**Keywords:** compulsory citizenship behavior (CCB), active-aggressive abusive supervision, passive-aggressive abusive supervision, emotional exhausion, perceived co-worker support

## Abstract

We conceptualize and examine an integrated model of compulsory citizenship behavior in the employees of the insurance sector. For this purpose, direct and indirect influences of job demands (active-aggressive and passive-aggressive abusive supervisions) are examined on the compulsory citizenship behavior. In so doing, the relevance of perceived support of coworkers as a job resource and emotional exhaustion as an underlying mechanism is investigated. Data were collected from 205 managerial level employees working in the insurance sector of a developing economy. Both aspects of abusive supervision have both direct and indirect effects through emotional exhaustion on compulsory citizenship behavior. Active-aggressive abusive supervision, coupled with low perceived support of the coworkers influences emotional exhaustion that culminates in increased compulsory citizenship behavior of employees. Interestingly, when perceived support of coworkers is high, the indirect link between active aggressive abusive supervision and compulsory citizenship behavior through emotional exhaustion decreases. For human resource managers, these findings imply that the proper background checks should be made before the recruitment so that employees with troubled past or tending to exhibit aggression can be screened. For line managers, these findings imply that coworkers can play a major role in curbing the compulsory citizenship behavior. Thus, managers should foster such organizational practices that can develop mutual trust and stronger relationship among coworkers so that coworkers can become the perfect source of psychological support.

## Introduction

Over the last few decades, organizational scholars have zeroed in on factors eliciting undesirable attitudes and the anomalous behavior of employees (Griffin and Lopez, 2005; Tourigny et al., 2013; Peng et al., 2019). These behavioral anomalies are often caused by contemporary work demands and pressures. Moreover, similar tendencies can be observed in the context of COVID-19 (Harris et al., 2007; Pradhan and Jena, 2018; Fouad, 2020; Restubog et al., 2020). In today’s environment, organizations are disposed to exploit their employees by asking them to perform extra duties which they end up performing due to pressure from the supervisors or top management (Zellars et al., 2002; Vigoda-Gadot, 2006, 2007; Fouad, 2020; Restubog et al., 2020). To accomplish organizational targets, supervisors might resort to negative behaviors toward their subordinates traditionally. In this context, citizenship behavior loses the voluntary aspect and becomes a matter of compulsion rather than a choice and it is termed as “compulsory organizational citizenship behavior,” hereafter compulsory citizenship behavior (Vigoda-Gadot, 2006; Zhang et al., 2011; Bolino et al., 2013). Thus, the concept of compulsory citizenship behavior represents a negative reflection of extra-role behavior–organizational citizenship behavior and has been distinguished as a distinct construct from the organizational citizenship behavior in the existing literature (Zhao et al., 2014).

Countries that have high power distance are most likely to have organizations with supervisors who influence their subordinates to a great extent (Peng and Zhao, 2012). These supervisors have an increased tendency to exhibit abusive behavior because of the high authority they exercise over their subordinates (Richard et al., 2018). This form of behavior results in higher levels of emotional exhaustion (Wu et al., 2012). Empirical evidence suggests that the frequency of negative supervisor behaviors is more prevalent in Asia than in the United States (Mackey et al., 2017). Therefore, in high-power distance countries having highly demanding industries such as insurance, the top management is considered to have final say in most, if not all decisions, and employees feel helpless when told to perform extra duties and indulge in extra-role behavior. The South Asian insurance industry has been significantly influenced not only by the macroenvironmental factors such as dynamic economic conditions and rapid technological change but also by the constant fluctuations in the company’s sales strategies (Bun, 2002; Sharma and Kaur, 2013; Rana and Javed, 2019). Under these conditions, employees experience stress and anxiety that further hinder their job performance and seriously impact social life (Gupta et al., 2014). The employees of the Pakistani insurance sector also experience immense pressure causing burnout, and subsequently high turnover intention and similar experiences are recorded in its neighboring country, India (Rana and Javed, 2019).

The dark side of organizational citizenship behavior, compulsory citizenship behavior, has received less attention ever since it was first identified. Some studies have very recently explored its consequences (Liu et al., 2017; He et al., 2018, He et al., 2020), whereas few scholars have looked at the mechanism through which this phenomenon occurs (Wu et al., 2018; Wang and Huang, 2019). Even though organizations might benefit from these extra duties performed by employees (Zhang et al., 2011), little is known as to when and how individuals opt for compulsory citizenship behavior (Zhao et al., 2013; Wu et al., 2018).

In the last 10 years, a handful of studies have explored the antecedents of abusive supervision but scholars have emphasized determining the outcome of the abusive form of supervision (Martinko et al., 2013; Tepper et al., 2017). Furthermore, Tepper et al. (2017, p. 132) have strongly urged researchers to research to untangle coping strategies and different processes that associate abusive supervision with work outcomes. Our conceptual model and its testing fill these important gaps. In this regard, we propose and empirically examine the link between two facets of abusive supervision (job demand) and compulsory citizenship behavior (extra-role performance) by integrating the underpinnings of job demands-resources (JD-R) theory. What is more, these associations are probed under the influence of emotional exhaustion (strain) as a mediator and in the presence of perceived coworker support (job resource) as a boundary condition or moderator. In so doing, we propose and test a moderated mediation model of abusive supervision and compulsory citizenship behavior.

Due to the innate scarcity of job resources as per the JD-R theory, the increase in job demands would cause a diminution of energy. This attenuation of work energy causes strain including emotional exhaustion, anxiety, hindrance stress and consequently, the poor in-role performance of employees (Bakker and Demerouti, 2014, 2017; Wu et al., 2018). It is argued that job resources can be extrinsically and intrinsically motivating (Langseth-Eide, 2019). As stated above, the occupational setting with higher job demands and constrained job resources like the insurance sector has the highest chances of strained work behaviors (Bakker et al., 2014; Langseth-Eide, 2019). This undesired buildup of demands necessitates employees to bank on resources and come out of the crisis. It implies that job-related resources can play a vital role in lessening the negative impact of job demands in the workplace (Bakker and Demerouti, 2017). In this regard, social support is an important resource that evinces a strong impact on burnout (Kim et al., 2018) and can also buffer the negative impact of work–family conflict and multitasking on employees’ turnover intention (Asghar et al., 2018, 2021). In organizations, employees consider supervisor support as more powerful and favorable than coworker support (Ng and Sorensen, 2008). However, in the absence of supervisor support, the employee might turn to their peers/coworkers, and lack of support from them might exacerbate the situation and cause emotional exhaustion in workers (Pradhan and Jena, 2018). Due to the abusive behavior of supervisors, subordinates may be reluctant to perform extra duties as the supervisors exhibit negative behaviors such as destructive leadership (Wu et al., 2018). On the contrary, since supervisors possess relevant resources and power within the organization, the workers might decide not to retaliate to stay in the supervisor’s good books and thus be compelled to perform extra duties involuntary, thereby engaging the compulsive citizenship behavior (Cropanzano and Mitchell, 2005; Wu et al., 2018).

According to Tummers and Bakker (2021), leadership-based constructs are frequently taken as job resources; however, a negative form of leadership (for example, abusive supervision, tyrannical leadership) is considered as a job demand. Previous studies have identified abusive supervision as a job demand (Wu et al., 2012) and perceived coworker’s support as an important job resource (Wu et al., 2012; Pradhan and Jena, 2018). It has been about two decades since the term abusive supervision was coined by Tepper (2000). Over time, the interest shown by scholars in this form of supervision has increased (Scheuer et al., 2016; Mackey et al., 2017; Pradhan and Jena, 2017; Wang and Huang, 2019). Recently, question marks have been raised about the dimensionality of abusive supervision, and researchers have been urged to investigate the multidimensional nature of abusive supervision (Martinko et al., 2013; Mackey et al., 2017). Two facets of abusive supervision, active-aggressive abusive supervision and passive-aggressive abusive supervision were identified (Mitchell and Ambrose, 2007; Decoster et al., 2014). Therefore, the lack of empirical evidence warrants further studies to probe into the dimensionality of abusive supervision (Mackey et al., 2017).

This manuscript is systematically organized into different sections. First, we develop a framework to further advance and refine the understanding of the process that links abusive supervision with employees’ compulsory citizenship behavior. This discussion is presented as a theoretical background and a hypotheses development section. Second, data collection procedures and details of instruments are given. This section follows the results section which provided the details of the data analysis and empirical tests. Last, the discussion of results, theoretical implications, practical implications, research limitations, and future research directions are provided.

### Theoretical Background and Hypothesis Development

#### Abusive Supervision as Job Demand

According to JD-R theory, job demands are “those physical, social, or organizational aspects of the job that require sustained physical or mental efforts and are therefore associated with certain physiological and psychological costs” (Demerouti et al., 2001, p. 501). Aggressive behavior is a multidimensional construct and has been defined by a number of researchers with no consensus over a precise definition, leading to a number of operational issues. A major issue related to aggressive behavior is about the appropriate classification of its subtypes. Broadly, this form of behavior can be expressed in either direct–indirect and active–passive form with various subtypes (Parrott and Giancola, 2007). Abusive supervision is a type of aggressive behavior, but the evidence pertaining to its multidimensionality is scarce. Abusive supervision is defined as “Sub-ordinates’ perceptions of the extent to which supervisors engage in the sustained display of hostile verbal and non-verbal behaviors, excluding physical contact” (Tepper, 2000, p. 178). Two distinct dimensions of abusive supervision were identified by Mitchell and Ambrose (2007), namely “passive-aggressive abusive supervision and active-aggressive abusive supervision.”

Active-aggressive abusive supervision encapsulates active acts of hostile behavior directed toward the subordinate (Mitchell and Ambrose, 2007). It has negative relationship with organizational citizenship behavior directed at individual (OCB-I), organizational citizenship behavior directed at organization (OCB-O), and leader–member exchange (LMX), but does not influence performance (Decoster et al., 2014). Furthermore, Baron and Neuman (1996) argued that the passive form of aggression is more prevalent in organizations than the active one. Passive-aggressive abusive supervision captures the passive acts of interpersonal abuse (Mitchell and Ambrose, 2007). This passive form of abuse includes “not giving credit for a job that requires a lot of effort,” “invasion of privacy,” and “blaming subordinate to save himself/herself from the embarrassment” (Mitchell and Ambrose, 2007, p. 1168). Passive-aggressive abusive supervision negatively affects OCB-O, OCB-I, LMX, and performance (Decoster et al., 2014). When employees are pressurized into performing extra duties, they are more likely to exhibit citizenship behaviors (Bolino et al., 2009). A study investigating the processes that create the supervisor–subordinate relationships showed that when supervisors perceived extra duties performed by subordinates as part of their in role duties, they do not consider it necessary to treat employees fairly (Simon et al., 2015).

Abusive supervision diminishes the tendency of subordinates to perform organizational citizenship behaviors. Subordinates who have experienced abusive forms of supervision are less likely to exhibit organizational citizenship behaviors than those whose supervisors are non-abusive. Several studies have reported a relationship between abusive supervision and organizational citizenship behavior (Zellars et al., 2002; Aryee et al., 2007; Xu et al., 2012; Decoster et al., 2014). However, there is a dearth of research regarding the link between abusive supervision and compulsory citizenship behavior except the following handful studies (Zhao et al., 2013; Wu et al., 2018) that have found a positive relationship between different forms of negative abusive supervision and compulsive citizenship behavior. Compulsory citizenship behavior is the antithesis of organizational citizenship behavior, therefore we expect both dimensions of abusive supervision to have positive impact on compulsory citizenship behavior. As per this rationale, we propose the positive relationships of active-aggressive and passive-aggressive abusive supervisions with compulsive citizenship behavior in the following hypotheses.

***Hypothesis 1_*a*_*** = *Active-aggressive abusive supervision is positively related with compulsory citizenship behavior.*
***Hypothesis 1_*b*_*** = *Passive-aggressive abusive supervision is positively related with compulsory citizenship behavior.*

#### Perceived Support of Coworker as a Job Resource

Job resources are “those physical, psychological, social, or organizational aspect of the job that may do any of the following: (1) be functional in achieving goals; (2) reduce job demands; (3) stimulate personal growth and development” (Demerouti et al., 2001, p. 501). Social support is a form of job resource and includes behaviors such as being helpful and showing respect to colleagues at work place, which has been used previously in a myriad of studies (Demerouti et al., 2001; Ng and Sorensen, 2008; Pradhan and Jena, 2018). The concept of social support is grounded on the “principle of reciprocity” and is further elucidated with the help of social exchange theory and JD-R theory (Emerson, 1976; Bakker and Demerouti, 2014, 2017; Cropanzano et al., 2017). With the help of social support, employees can perform their tasks effectively in the organization (Ng and Sorensen, 2008) and it can also be instrumental in dealing with job stress (Terry et al., 1993; Wu and Hu, 2009). Due to finite resources, employees have to ensure that in their time of need they receive support from their peers. This happens because employees would expect their diminished resources to be replenished through social support (Halbesleben and Bowler, 2007).

The concept of social support can further be explained with the help of buffering hypothesis. According to this proposition, social support can be defined as a “mechanism through which interpersonal relationships presumably buffer one against a stressful environment” (Cohen and McKay, 1984, p. 253). The buffering hypothesis posits that people with social support can experience a reduced impact on their health and overall well-being as compared with those who are without one (Cohen and McKay, 1984). Social support has been bifurcated into perceived support and received support. Out of both these forms perceived support, which includes perceived support of supervisor and perceived support of coworkers has been repeatedly found to be associated with the well-being of an individual (Haber et al., 2007; Ng and Sorensen, 2008). For instance, Duffy et al. (2002) investigated social undermining in organizations, and both supervisor undermining and coworker undermining were found to cause counterproductive behaviors and somatic complaints in employees. These effects were found to be stronger when the supervisor assumed the dual roles of both supporter and underminer. Nevertheless, the crossdomain effects somewhat supported the notion of buffering given that the deleterious effect of the supervisor’s undermining on somatic complaints was alleviated by the coworker’s support.

Perceived coworker’s support is defined as “the extent to which employees believe their coworkers are willing to provide them with work-related assistance to aid in the execution of their service-based duties” (Susskind et al., 2003, p. 181). Coworker’s support can moderate the relationship between abusive supervision and emotional exhaustion (Wu et al., 2012; Pradhan and Jena, 2018). A study conducted on healthcare professionals showed that high levels of perceived coworker’s support can lessen the negative impact of abusive supervision on emotional exhaustion (Pradhan and Jena, 2018). However, Wu and Hu (2009) reported a finding contrary to the proposed hypothesis as abusive supervision’s impact on emotional exhaustion was enhanced when the perceived coworker’s support was high. This phenomenon was explained with the help of reverse buffering effect and the ceiling effect. In some instances, peers albeit showing their support, tend to enhance the victim’s negative feeling toward the supervisor, thus activating the reverse buffering effect. An alternate explanation is also provided by a ceiling effect. According to the ceiling effect, employees receiving less support from their coworkers already achieve a certain stagnation in relation to their emotional exhaustion. Beyond that “ceiling point,” any increase in the coworker’s support exacerbates the situation (Wu and Hu, 2009). Thus, based on JD-R theory and empirical evidence, the following hypotheses are postulated.

***Hypothesis 2_*a*_*** = *Perceived support of coworkers moderates the relationship between active-aggressive abusive supervision and emotional exhaustion such that the positive relationship between active-aggressive abusive supervision and emotional exhaustion is more strengthened when perceived support of coworkers is low.*
***Hypothesis 2_*b*_*** = *Perceived support of coworkers moderates the relationship between passive-aggressive abusive supervision and emotional exhaustion such that the positive relationship between passive-aggressive abusive supervision and emotional exhaustion is more strengthened when perceived support of coworkers is low.*

#### Abusive Supervision and Emotional Exhaustion

Abusive behaviors of supervisors may induce psychological discomfort in subordinates, which includes depression, anxiety, and emotional exhaustion (Tepper, 2000). It is important to emphasize that emotionally exhausted employees usually feel that they are deficient of adaptive resources which limits the extent to which they can do extra for their jobs (Halbesleben and Buckley, 2004, p. 859). Abusive supervision has a significant positive relationship with emotional exhaustion (Tepper, 2000; Yagil, 2006; Tepper et al., 2007; Aryee et al., 2008; Breaux et al., 2008; Wu and Hu, 2009; Whitman et al., 2014; Scheuer et al., 2016; Pradhan and Jena, 2018). Furthermore, results of a metaanalysis supported a modest association of abusive supervision with emotional exhaustion (Mackey et al., 2017). One of the studies showed that both facets of active-aggressive and passive-aggressive supervision are expected to yield similar findings as a composite dimension of abusive supervision (Decoster et al., 2014).

Tepper et al. (2017) proposed two distinct performance enhancing and undermining pathways that lead abusive supervision to employee performance. The performance undermining path emanating from abusive supervision triggers stronger impact on job performance than the performance enhancing pathway. Thus, abusive supervision can impede employee performance if it involves negative reciprocity, compromise on team dynamics, and negative role modeling and resource depletion. This depletion of resources due to work strain–emotional exhaustion decreases job performance of employees (Wright and Cropanzano, 1998; Bakker and Heuven, 2006; Karatepe, 2013). Employees, being the constant part of abusive supervisor–subordinate relationship, exhaust their resources resulting in emotional exhaustion. After becoming emotionally drained the subordinates lack the energy to concentrate on their job with full enthusiasm (Aryee et al., 2008).

Previous studies have reported that the link between abusive supervision and citizenship behaviors usually involves a mediator (Xu et al., 2012; Decoster et al., 2014). For instance, burnout has negative association with OCB (Liang, 2012), whereas emotional exhaustion, a type of burnout, also has a negative impact on organizational citizenship behavior (Chiu and Tsai, 2006; Chang et al., 2007; Liang, 2012) including OCB-Organization (Cropanzano et al., 2003; Chang et al., 2007; Tourigny et al., 2013) and OCB-Individual (Chang et al., 2007). Role of emotional exhaustion as a mediator has been probed before. For instance, the association between job demand and health problems is mediated by burnout (Schaufeli and Bakker, 2004). Emotional exhaustion also has a mediating effect on the relationship between job demands and negative organizational outcomes (Karatepe, 2013), customer and employee incivility (Van Jaarsveld et al., 2010), and surface acting and breaking character (Grandey, 2003). Similarly, the link between abusive supervision and counterproductive work behavior was mediated by the emotional exhaustion (Akram et al., 2019). As per the above discussion, this study expects emotional exhaustion to mediate the relationship between both facets of abusive supervision and compulsory citizenship behavior as proposed in the following hypotheses.

***Hypothesis 3_*a*_*** = *The positive relationship between active-aggressive abusive supervision and compulsory citizenship behavior is mediated by emotional exhaustion.*
***Hypothesis 3_*b*_*** = *The positive relationship between passive-aggressive abusive supervision and compulsory citizenship behavior is mediated by emotional exhaustion*.

#### Collective Role of Perceived Coworker Support as a Boundary Condition and Emotional Exhaustion as an Underlying Mechanism

Various forms of job demands, like abusive supervision, workload demands (Wu and Hu, 2009), and work overload (Karatepe, 2013) cause depletion of resources (Schaufeli and Bakker, 2004). To establish a good relationship with their supervisors, the subordinates are expected to adjust their emotions (Tepper et al., 2007). This form of adjustment would require subordinates to take extra efforts resulting in the loss of resources and becoming emotionally exhausted (Grandey, 2000; Karatepe, 2013). These resources are further diminished in the presence of low perceived coworker’s support (Pradhan and Jena, 2018). When faced with these diminished resources, employees tend to invest remaining of their energy in citizenship behaviors which would reap future benefit at the cost of the duties that they are bound to perform, which is in-role performance (Halbesleben and Bowler, 2007). Citizenship behaviors are directed toward individuals (supervisors and peers) and organization as a whole. In the circumstances where the employee is already experiencing diminished emotional resources, the coworkers might step in by showing support and demanding less of the extra duties. Perhaps then the individuals might end up exhibiting less compulsory organizational citizenship behaviors and vice versa. Therefore, on the basis of aforementioned discussion, the following hypotheses are proposed to examine the first stage moderated (perceived coworker support) mediation (emotional exhaustion) for the associations of active-aggressive and passive-aggressive abusive supervisions with compulsory citizenship behavior.

**H4_*a*_** = *The indirect effects from active-aggressive abusive supervision to compulsory citizenship behavior are moderated by perceived coworker support through emotional exhaustion and these effects are more strengthened when perceived support of coworker is low rather than high.*
**H4_*b*_** = *The indirect effects from passive-aggressive abusive supervision to compulsory citizenship behavior are moderated by perceived coworkers’ support through emotional exhaustion and these effects are more strengthened when perceived support of coworker is low rather than high*.

**FIGURE 1:**
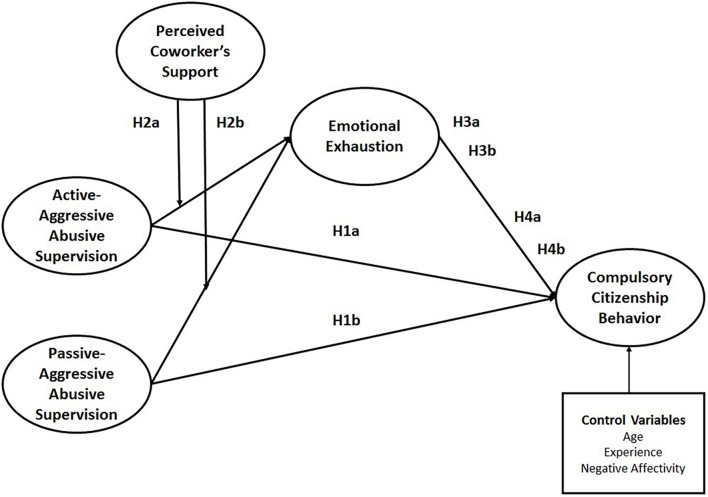
Conceptual framework.

The conceptual framework representing all of the aforementioned hypotheses is presented in Figure 1. This conceptual framework provides an integrated model of compulsory citizenship behavior and illustrates how work demands can influence extra-role performance through strain in the presence of job resource as a boundary condition. For this purpose, it is conceptualized that two different facets of abusive supervision (active-aggressive abusive supervision and passive-aggressive abusive supervision) as work demands can influence compulsory citizenship behavior through strain (emotional exhaustion), and this relationship can be moderated by job resource (perceived coworker’s support).

## Materials and Methods

### Sample and Data Collection Procedures

The unit of analysis and target respondents for this research were individuals. The sampling technique adopted for data collection was the purposive sampling which involved selecting individuals working with insurance companies at managerial posts with the experience of at least 3 years within the same sector. In this study, crosssectional research design was adopted to collect data through self-administered surveys. Before the survey was administered to potential respondents, 20 individuals working in insurance sector having diverse academic background, job experience, and position in the company’s hierarchy for conducting pretest of the questionnaire were selected. Overall, feedback indicated that participants were able to comprehend and interpret the questionnaires correctly.

There are 41 general insurance corporations operating in Pakistan. Majority of these companies were contacted over phone to seek permission for data collection. Finally, nine companies gave their permission to collect data. For this purpose, initial meetings were scheduled with company representatives to brief them about the aim of this research. The anonymity of the respondents was guaranteed given that the nature of the questionnaire was such that respondents would have felt reluctant in filling out the survey. With the help of the contact person in every organization for this study, employees with more than 3 years of experience in the industry occupying managerial level post in the organization were identified. Questionnaires were administered to respondents using pencil and paper survey method, similar to the that data was collected in 2019. Each organization was visited at least two to three times with respondents being given an option to either fill out the survey form immediately or at the time of their convenience. A total of 212 questionnaires were returned by the respondents out of which seven were rejected for having missing values. Therefore, the final usable responses were 205. The majority (around 92%) of the respondents were men. Married employees comprised 80% of the total respondents. As far as the academic qualification is concerned, approximately 64% of the respondents had a post-graduate degree and the remaining 36% held an undergraduate degree.

### Research Instruments

Two dimensions of abusive supervision (active and passive aggressive) were extracted by Mitchell and Ambrose (2007) from the original scale of abusive supervision developed by Tepper (2000). Each dimension comprises of five items and were measured on a five-point agreement scale. Sample items of active-aggressive abusive supervision include, *“My supervisor tells me I’m incompetent”* and “*My supervisor tells me my thoughts or feelings are stupid*.” Passive-aggressive abusive supervision’s sample items include, “*My supervisor doesn’t give me credit for jobs requiring a lot of effort*” and “*My supervisor blames me to save himself/herself embarrassment*.” A five-item scale of compulsory citizenship behavior developed by Vigoda-Gadot (2006) was used to measure by frequency-based scale points with 1 depicting “Never” and 5 indicating “Always.” Few of the items include, *“The management in this organization puts pressure on employees to engage in extra-role work activities beyond their formal job tasks”* and *“There is social pressure in this organization to work extra hours, beyond the formal workload and without any formal rewards.”* Perceived support of coworkers was gauged by three items adopted by Wu and Hu (2009) and was developed by Staw et al. (1994). It was gauged on five-point Likert or agreement scale. Sample items include, *“I and my coworkers share news about important things that happen at the organization,”* and *“My coworkers give me the help I need to do my job.”* The five items of emotional exhaustion were adopted from MBI-GS and has originally been measured on a seven-point Likert scale; with 0 indicating “never” to 6 indicating “everyday” frequency rating scale (Schaufeli et al., 1996). However, studies have also used the five-point agreement scale with 1 indicating “strongly disagree” to 5 depicting “strongly agree” (Lloyd et al., 2015). Sample items include, *“I feel used up at the end of the workday,” “Working all day is really a strain for me.”*

Past studies have shown that respondent’s ability to perform extra duties can be influenced by their age, experience (Zhao et al., 2013), and negative affectivity (Tepper et al., 2006; Wu and Hu, 2009). Therefore, we controlled for these factors in our research. Age and industry experience were measured in years. Negative affectivity was gauged through PANAS scale developed by Watson et al. (1988). Target respondents were requested to rate five emotions “nervous, afraid, upset, irritable, and distressed” on the scale of 1 (very slightly or none at all) to 5 (extremely). A complete list of items of each construct is presented in Annexure A. The Ethics Committee of the Lahore School of Economics as the constituent committee of Committee for Advanced Studies & Research has given the ethical approval of this study.

Two software namely SPSS (version 20) and AMOS (version 23) were used for the screening of the data and conducting analyses. Hayes Process macro (version 3.2) was used for testing hypotheses 2, 3, and 4.

## Results

The test for skewness and kurtosis was performed for determining normality. If the value of skewness is greater than ± 2 than the normality of the data cannot be established. Similarly, for the kurtosis, the desired range is ± 7 (West et al., 1995). The results (reported in Supplementary Material section) indicate that all values were within the prescribed range, hence there were no issues of normality.

### Common Method Variance

Common method variance (CMV) is “variance that is attributable to the measurement method rather than to the constructs the measures represent” (Podsakoff et al., 2003, p. 879). Two different *post hoc* statistical tests, Harman’s single-factor test, and common latent factor were used to assess whether or not CMV is an issue. The results from Harman’s single factor test showed that a single factor only attributed to 40.85% of the variance, which is less than the cutoff value of 50% (Podsakoff and Organ, 1986).

For the common latent factor test, two different models (base measurement model and measurement model with the latent construct) were compared. Ideally, the difference between the two should be less than 0.20 (Gaskin, 2016). Results (reported in Supplementary Material section) showed that measurement error was not a cause of concern.

### Measurement Model Results

A set of confirmatory factor analyses were conducted using AMOS (version 23) to determine whether respondents were successfully able to distinguish all constructs in the hypothesized model. For this purpose, sequential Chi-square difference test was performed with five alternative measurement models. The five-factor model showed substantial improvement in χ^2^ (four-factor model, A, Δχ^2^ = 79.73**^∗∗∗^**, *P* < 0.01; four-factor model, B, Δχ^2^ = 163.577**^∗∗∗^**; three-factor model, Δχ^2^ = 199.525**^∗∗∗^,** *P* < 0.01; two-factor model, Δχ^2^ = 394.736**^∗∗∗^**, *P* < 0.01; single-factor model, Δχ^2^ = 619.875**^∗∗∗^**, *P* < 0.01). CFI and IFI of the five-factor model were relatively better than the alternative models and above the cutoff value of 0.90 (Hair et al., 2010). Overall, results suggested that the five-factor model (hypothesized model) fits the data relatively better than all the remaining nested models. According to Hair et al. (2010), factor loading of each item of a construct should be at least ≥ 0.50. Therefore, all items with factor loadings of less than 0.50 were dropped from the model.

Fornell and Larcker (1981) suggested that the convergent validity holds for the latent construct if the average value extracted (AVE) turns out to be greater than 0.5. To establish discriminant validity of constructs, the value of AVE should be greater than the value of the shared variance. For the reliability, two different diagnostic measures Cronbach’s Alpha and composite reliability were used with values of ≥ 0.70 generally considered reliable (Hair et al., 2010). The results indicate that there were no reliability and validity concerns in the model. The measurement model results are reported in Supplementary Material section.

### Bivariate Analysis

The correlations among emotional exhaustion and both facets of abusive supervision (active-aggressive and passive-aggressive) were 0.556 and 0.567, respectively (both *P* < 0.01). Although the correlation between compulsory citizenship behavior and emotional exhaustion was strong and positive (*r* = 0.607, *P* < 0.01), perceived support of coworkers had positive but weak correlation with EE (*r* = 0.178, *P* < 0.05). Both active-aggressive (*r* = 0.60, *p* < 0.01) and passive-aggressive abusive supervision (*r* = 0.59, *P* < 0.01) were significantly correlated with compulsory citizenship behavior. Pearson correlations coefficients are reported in Table 1

**TABLE 1:** Summary statistics and correlation of variables

	**Mean**	***SD***	**1**	**2**	**3**	**4**	**5**	**6**	**7**	**8**
1. Emotional exhaustion	2.57	1.148	–							
2. Compulsory citizenship behavior	2.93	1.015	0.607**	–						
3. Perceived coworker’s support	3.82	0.831	0.178*	0.087	–					
4. Passive-aggressive abusive supervision	2.14	0.809	0.556**	0.598**	0.189**	–				
5. Active-aggressive abusive supervision	2.23	0.955	0.567**	0.600**	0.096	0.765**	–			
6. Negative affectivity	2.21	0.832	0.095	0.387**	0.077	0.284**	0.245**	–		
7. Experience	10.9	6.55	–0.041	–0.084	–0.043	–0.047	–0.059	−0.089	–	
8. Age	36.7	6.82	–0.033	–0.066	–0.024	–0.022	–0.025	−0.037	0.883**	–

**Correlation is significant at the 0.01 level (2-tailed).

*Correlation is significant at the 0.05 level (2-tailed).

### Hypotheses Testing

Hypotheses 1_a_ and 1_b_ were tested using multiple linear regression in SPSS (version 20). Tables 2, 3 show regression results with active-aggressive abusive supervision and passive-aggressive abusive supervision, respectively. Regression results show that both active-aggressive abusive supervision (β = 0.538, *p* < 0.01) and passive-aggressive abusive supervision (β = 0.530, *p* < 0.01) significantly predicted compulsory citizenship behavior in employees. Therefore, Hypotheses 1_a_ and 1_b_ were supported.

**TABLE 2:** Regression analysis with active-aggressive abusive supervision.

	**Dependent variable**	
**Variable(s)**	**Compulsory citizenship behavior**	
	***Step 1***	***Step 2***
**Control variables**		
*Negative affectivity*	0.384***	0.256***
*Experience*	−0.020 (n.s)	0.040 (n.s)
*Age*	−0.033 (n.s)	−0.078 (n.s)
**Independent variable**		
*Active-aggressive abusive supervision*		0.538***
***R* ^2^**	0.153	0.424
**Adjusted *R*^2^**	0.140	0.412
***F***	12.085***	36.731***
**Δ*R*^2^**	–	0.271
**ΔF**	–	93.909***

***p < 0.01.

**TABLE 3:** Regression analysis with passive-aggressive abusive supervision.

	**Dependent variable**	
**Variable(s)**	**Compulsory citizenship behavior**	
	***Step 1***	***Step 2***
**Control variables**		
*Negative affectivity*	0.384***	0.235***
*Experience*	−0.020 (n.s)	0.05 (n.s)
*Age*	−0.033 (n.s)	−0.050 (n.s)
**Independent variable**		
*Passive-aggressive abusive supervision*		0.530***
***R* ^2^**	0.153	0.411
**Adjusted *R*^2^**	0.140	0.399
***F***	12.085***	34.895***
**ΔR^2^**	–	0.258
**ΔF**	–	87.688***

***p < 0.01.

Hypotheses 2_a_ and 2_b_ were tested using model 1 of the Hayes process macro in SPSS (version 20). The results for H2_a_ shown in Table 4 indicates that active-aggressive abusive supervision has a significant impact on emotional exhaustion (β = 0.602, *P* < 0.01), whereas the interaction effect of active-aggressive abusive supervision and perceived the coworker’s support on emotional exhaustion also came out to be statistically significant (β = −0.1331, *P* < 0.10).

**TABLE 4:** Moderation results with active-aggressive abusive supervision.

**Relationship**	**Standardized coefficient β**	**SE**	***t***	***p*-value**
*Active-aggressive abusive supervision **→**Emotional exhaustion*	0.6023***	0.0625	9.63	0.000
*Perceived coworker’s support **→** Emotional exhaustion*	0.1272**	0.0572	2.22	0.027
*Active-aggressive abusive supervision* **X** *Perceived coworker’s support **→** Emotional exhaustion*	−0.1331*	0.706	–1.8855	0.060

***p < 0.01; **p < 0.05; *p < 0.10.

Similarly, for H2_b_, the results in Table 5 show that passive–aggressive abusive supervision has a positive impact on emotional exhaustion (β = 0.5803, *p* < 0.01), and the interaction effect of passive-aggressive abusive supervision and perceived coworker’s support on emotional exhaustion was also statistically significant (β = −0.1066, *P* < 0.10). Albeit statistically significant, the interaction effects for both H2_a_ and H2_b_ were weak. Penney and Spector (2005) in their research study on counterproductive work behavior, supported moderation hypothesis with 0.10 as significance criterion.

**TABLE 5:** Moderation results with passive-aggressive abusive supervision.

**Relationship**	**Standardized coefficient β**	**SE**	***t***	***p*-value**
*Passive-Aggressive Abusive Supervision **→** Emotional Exhaustion*	0.5803***	0.0633	9.1622	0.000
*Perceived Coworker’s Support **→** Emotional Exhaustion*	0.077	0.0591	1.33	0.1941
*Passive-Aggressive Abusive Supervision* ***X*** *Perceived Coworker’s Support **→** Emotional Exhaustion*	−0.1066*	0.0626	–1.7063	0.0895

***p < 0.01; *p < 0.10.

Table 6 shows the impact of both active-aggressive and passive-aggressive abusive supervision on emotional exhaustion across different levels of perceived coworker’s support. For both forms of abusive supervision, when the perceived coworker’s support is low the impact on emotional exhaustion is more strengthened, whereas high support lessens their impact on emotional exhaustion. Thus, both Hypotheses 2_a_ and 2_b_ were supported as shown in Table 6.

**TABLE 6:** Moderation results with active-aggressive and passive-aggressive abusive supervision for different levels of coworker’s support.

**Perceived coworker’s support**	**Effect of active aggressive abusive supervision on emotional exhaustion**	**Effect of passive aggressive abusive supervision on emotional exhaustion**	***p*-value**
Low	0.7353***	0.6868***	0.00
Med	0.5729***	0.5568***	0.00
High	0.4717***	0.4757***	0.00

***p < 0.01.

Hypotheses 3_a_ and 3_b_ were tested using model 4 of the Hayes process macro. The mediation analysis involved the computation of direct and indirect effects from active aggressive-abusive supervision to compulsory citizenship behavior through emotional exhaustion for testing H3_a_. In a similar vein, H3_b_ was tested by calculating direct and indirect effects from passive–aggressive abusive supervision to compulsory citizenship behavior through emotional exhaustion.

Results of mediation analysis are shown in Tables 7, 8. The direct effects from both active-aggressive abusive supervision and passive-aggressive abusive supervision to compulsory citizenship behavior were statistically significant. The standardized indirect effect from active-aggressive abusive supervision to compulsory citizenship behavior through emotional exhaustion was statistically significant (Indirect effect = 0.2325, Boot CI [0.1538, 0.3241]). Similarly, the indirect effect from passive–aggressive abusive supervision to compulsory citizenship behavior through emotional exhaustion was also statistically significant (Indirect effect = 0.2338, Boot CI [0.1490, 0.3384]).

**TABLE 7:** Mediation results with direct effects.

**Relationship**	**Direct effects**	**SE**	***t***	***p*-value**
*Active-aggressive abusive supervision **→** Compulsory citizenship behavior*	0.3001***	0.0603	4.978	0.000
*Passive-aggressive abusive supervision **→** Compulsory citizenship behavior*	0.2884***	0.0607	4.7491	0.000

***p < 0.01.

**TABLE 8:** Mediation results with indirect effects.

**Relationship**	**Indirect effects**	**BootLLCI**	**BootULCI**
*Active-aggressive abusive supervision **→** Emotional exhaustion **→** Compulsory citizenship behavior*	0.2325	0.1538	0.3241
*Passive-aggressive abusive supervision **→** Emotional exhaustion **→** Compulsory citizenship behavior*	0.2338	0.1490	0.3384

Zhao et al. (2010) suggested that if the mediated effect (a × b) along with the direct effect (c) is significant and are in the same direction then the mediation is complementary in nature. Based on this classification, both H3_a_ and H3_b_ are supported with the type of mediation to be complementary in nature.

Hypotheses 4_a_ and 4_b_ were tested through model 7 of Hayes process macro (version 3.2). Table 9 shows index of moderated mediation for H4_a_ and H4_b_. The results show that for H4_*a*_, the index of moderated mediation was statistically significant because the confidence interval does not include zero. It indicates that the indirect effect of active-aggressive abusive supervision on compulsory citizenship behavior through emotional exhaustion is negatively moderated by the perceived coworker’s support. However, for H4_b_ the index was statistically insignificant, and hence the results cannot be further interpreted. Therefore, H4_b_ was not accepted.

**TABLE 9:** Index of moderated mediation.

**Relationship**	**Moderator**	**Index**	**Boot SE**	**Boot LLCI**	**Boot ULCI**
*Active-aggressive abusive supervision **→** Emotional exhaustion **→** Compulsory citizenship behavior*	Perceived coworker’s support	−0.0546	0.0262	−0.1068	–0.0034
*Passive-aggressive abusive supervision**→** Emotional exhaustion **→** Compulsory citizenship behavior*	Perceived coworker’s support	−0.0449	0.0272	−0.1021	0.0066

For three different levels of perceived coworker’s support, the conditional indirect effects are shown in Table 10. These levels were generated by process macro on the basis of 16th, 50th, and 84th percentile for low, mean, and high values, respectively. The results show that as the level of perceived coworker’s support increases, the employees’ tendency to engage in compulsory citizenship behavior decreases. Therefore, Hypotheses 4_a_ was supported as shown in Table 10.

**TABLE 10:** Conditional indirect effects from active-aggressive abusive supervision to compulsory citizenship behavior for different levels of perceived coworker’s support.

**Moderator**	**Level**	**Conditional indirect effect**	**Boot SE**	**Boot LLCI**	**Boot ULCI**
Perceived coworker’s support	**Low**	0.3016	0.0519	0.2079	0.4122
	**Mean**	0.2350	0.0418	0.1594	0.3246
	**High**	0.1935	0.0469	0.1146	0.2983

## Discussion

The aim of this research was to expand the current understanding on the formation of the dark side of organizational citizenship behavior through the JD-R theory. To study this complex phenomenon, the indirect impact of job demands on compulsory citizenship behavior through work strain was investigated in the presence of the social support as a boundary condition. As discussed previously, there is a dearth of studies that have empirically tested facets of abusive supervision regarding compulsive citizenship behavior. Similarly, compulsory citizenship behavior has received less attention (Vigoda-Gadot, 2007; Zhao et al., 2014). The results of this study showed that the presence of low perceived coworkers’ support reinforces the positive impact of both facets of abusive supervision on emotional exhaustion of employees. In addition, these aspects of abusive supervision have both direct as well as indirect effects (through emotional exhaustion) on compulsory citizenship behavior. Active-aggressive abusive supervision, coupled with low perceived coworkers’ support, causes emotional exhaustion that culminates in increased level of compulsory citizenship behavior. When employees received more support from their coworkers; the indirect link between active-aggressive abusive supervision and compulsory citizenship behavior through emotional exhaustion is abated. However, similar conditional indirect effects were not present for passive–aggressive abusive supervision. Moderated mediation results further provided interesting insights regarding how coworkers play a major role in curbing the compulsory citizenship behavior in employees. It has been found that lack of support from coworkers can further amplify emotional exhaustion in employees, related to both active and passive forms of abusive supervision. Interestingly, even when coworkers are very supportive, employees continue to be emotionally drained. However, as the abusive form of supervision increases, the employee with more support from coworkers would still feel less emotionally exhausted compared with those receiving less or no coworker support.

### Theoretical Implications

This study has made an important contribution by explaining the impact of different supervisory behaviors (active-aggressive and passive aggressive abusive supervision) on the dark side of employee performance through the lens of JD-R theory. Second, this investigation has been made by keeping in view how the emotional exhaustion (work strain) as an underlying mechanism influence the relationships of job demands and resources with compulsory citizenship behavior. In this manner, this study makes an important contribution by examining the direct link of two-dimensional supervisory behavior (active-aggressive and passive-aggressive abusive supervisions) with the dark side of the employee in-role performance (compulsory citizenship behavior). This study has used scales of both dimensions of abusive supervision adopted by Mitchell and Ambrose (2007), and Tepper (2000) to establish the multidimensional antecedents of compulsory citizenship behavior. The results from this research offers compelling evidence for the multidimensionality of abusive supervision. The findings further exhibited the relevance of JD-R theory regarding the multidimensional antecedents of compulsory citizenship behavior. Both forms of abusive supervision as job demands cause employees to be emotionally drained and also enhances their tendency to perform extra duties compulsorily. This evidence provides support to the notion that organizational citizenship behavior loses its voluntary aspect when job demands such as abusive supervision coerce their subordinates to render extra duties (Vigoda-Gadot, 2007; Zhao et al., 2014).

### Practical Implications

First, employees who fear a major backlash in failure of doing more on the job would succumb to this pressure and are more prone to this form of behavior (Zhao et al., 2013). For human resource managers, these findings imply to protect the employees from any mistreatment from their supervisors. Hence, it has become of paramount importance to make proper background check *a priori* to curb this menace. Those with a troubled past or having a tendency to exhibit aggression should not be hired (Pradhan and Jena, 2018). Second, since performance of compulsory organizational citizenship behavior is linked with a multitude of negative consequences such as employee silence and occupational stress (Vigoda-Gadot, 2007) amongst many others, it is pivotal for organizations to change perceptions of employees pertaining to extra duties from being compulsory to voluntary ones. One way of accomplishing this is by taking care of the well-being of their employees and showing support in their time of need (Peng and Zhao, 2012).

With respect to job resource, this study finds that low perceived coworker’s support can further amplify emotional exhaustion in employees, which is caused due to active and passive forms of abusive supervision. Interestingly, even when coworker’s support is high, the employee continues to be emotionally drained. However, as the abusive form of supervision increases, the employee with more support from coworker would still feel less emotionally exhausted compared with the one receiving less or no coworker support. Thus, coworkers act as an important job and emotional resource to deal with increased level of work demand. The findings of this study further imply that coworkers can play a major role in dealing with the compulsory citizenship behavior of employees for managers. Active-aggressive form of abusive supervision results in emotional exhaustion in employees, which further culminates in compulsory citizenship behavior, albeit the degree of those extra duties performed is dependent on support from the coworkers. In a situation where peers are more supportive, emotionally drained employees will still perform compulsory citizenship behavior, but to a lesser extent. Hence, the organizational managers should foster such organizational practices that can develop mutual trust and stronger relationship among coworkers as coworkers can be the perfect source of psychological support (Mathieu et al., 2019).

### Limitations and Future Research Directions

Every research work has a few shortcomings, and this research work is no exception. This study has relied on the crosssectional research design, but future studies can adopt longitudinal research design to capture the formation of compulsory citizenship behavior. There are several avenues for the future research that demands attention. This research can be replicated in other industries and cultures with both high and low power distance to validate and compare current findings. Also, other forms of strain and job demands can be utilized to understand the antecedents of compulsory citizenship behavior. One possible avenue for future research could be to investigate the role of polychronicity on compulsory citizenship behavior. Previous works have indicated polychronicity to have a significant impact on life satisfaction, work-family conflict, and procrastination behavior (Asghar et al., 2020; Gull et al., 2021; Xiaolong et al., 2021). Future studies can also probe into the role of other job resources in lessening undesirable impact of job demands on the wellbeing of employees. This work was conducted in the physical work setting prior to the pandemic, but the same model can be extended to understand additional job demands in the remote work setting. For now, this study has demonstrated an integrated model of compulsory citizenship behavior by investigating when and how active-aggressive and passive-aggressive abusive supervisions as job demands can influence compulsory citizenship behavior.

## Data Availability Statement

The raw data supporting the conclusions of this article will be made available by the authors, without undue reservation.

## Ethics Statement

The studies involving human participants were reviewed and approved by Ethics Committee of Lahore School of Economics. The patients/participants provided their written informed consent to participate in this study.

## Author Contributions

All authors listed have made a substantial, direct, and intellectual contribution to the work, and approved it for publication.

## Conflict of Interest

The authors declare that the research was conducted in the absence of any commercial or financial relationships that could be construed as a potential conflict of interest.

## Publisher’s Note

All claims expressed in this article are solely those of the authors and do not necessarily represent those of their affiliated organizations, or those of the publisher, the editors and the reviewers. Any product that may be evaluated in this article, or claim that may be made by its manufacturer, is not guaranteed or endorsed by the publisher.
