# Supplementary material for: Tracing an Unyielding Work Compulsion: A Moderated Mediation Model of Abusive Supervision and Compulsory Citizenship Behavior
Source: Front Psychol. 2021 Nov 29;12:746823. doi: 10.3389/fpsyg.2021.746823 (PMC8666692; doi:10.3389/fpsyg.2021.746823)
Supplement: Supplementary file 1 [file Table_1.DOCX]

Supplementary Material

**Study Constructs and Items**

**Active- Aggressive Abusive Supervision** (Tepper, 2000; Mitchell & Ambrose, 2007)

1. My supervisor ridicules me
2. My supervisor tells me my thoughts or feelings are stupid
3. My supervisor puts me down in front of others
4. My supervisor makes negative comments about me to others
5. My supervisor tells me I’m incompetent

**Passive- Aggressive Abusive Supervision** (Tepper, 2000; Mitchell & Ambrose, 2007)

1. My supervisor invades my privacy
2. My supervisor doesn't give me credit for jobs requiring a lot of effort
3. My supervisor blames me to save himself/herself embarrassment
4. My supervisor breaks promises he/she makes
5. My supervisor lies to me

**Emotional Exhaustion** (Schaufeli et al., 1996)

1. I feel emotionally drained from my work
2. I feel used up at the end of the workday
3. I feel tired when I get up in the morning and have to face another day on the job
4. Working all day is really a strain for me
5. I feel burned out from my work

**Perceived Coworker’s Support** (Staw et al., 1994 ;Wu and Hu , 2009)

1. My coworkers give me the help I need to do my job
2. I and my coworkers share news about important things that happen at the organization
3. I and my coworkers stick together

**Compulsory Citizenship Behavior** (Vigoda-Gadot ,2006)

1. The management in this organization puts pressure on employees to engage in extra-role work activities beyond their formal job tasks
2. There is social pressure in this organization to work extra hours, beyond the formal workload and without any formal rewards
3. I feel that I am expected to invest more effort in this job than I want to and beyond my formal job requirements
4. I feel that I am forced to help other coworkers beyond my formal obligations and even when I am short on time or energy
5. I feel that I am forced to assist my supervisor against my will and beyond my formal job obligations

**Negative Affectivity**  (Watson, Clark & Tellegen , 1988)

1. Nervous
2. Afraid
3. Upset
4. Irritable
5. Distressed

**Table A: Skewness and Kurtosis**

| **Constructs** | **Skewness** | **Kurtosis** |
| --- | --- | --- |
| Emotional Exhaustion | 0.293 | -1.133 |
| Compulsory Citizenship Behavior | -0.003 | -0.924 |
| Perceived Coworker's Support | -0.625 | -0.431 |
| Passive-Aggressive Abusive Supervision | 0.674 | -0.117 |
| Active-Aggressive Abusive Supervision | 0.897 | 0.405 |

**Table B: Harman’s Single Factor Test for Common Method Variance**

| **Total Variance Explained** | | | | | | |
| --- | --- | --- | --- | --- | --- | --- |
| **Factor** | **Initial Eigenvalues** | | | **Extraction Sums of Squared Loadings** | | |
|  | Total | % of Variance | Cumulative % | Total | % of Variance | Cumulative % |
| 1 | 7.450 | 43.823 | 43.823 | 6.946 | 40.857 | 40.857 |
| 2 | 2.233 | 13.133 | 56.956 |  | | |
| 3 | 1.467 | 8.629 | 65.585 |  |  |  |
| 4 | 1.080 | 6.351 | 71.936 |  |  |  |
| 5 | .711 | 4.182 | 76.118 |  |  |  |
| 6 | .672 | 3.952 | 80.070 |  |  |  |
| 7 | .536 | 3.155 | 83.225 |  |  |  |
| 8 | .511 | 3.005 | 86.230 |  |  |  |
| 9 | .423 | 2.490 | 88.720 |  |  |  |
| 10 | .352 | 2.071 | 90.791 |  |  |  |
| 11 | .310 | 1.824 | 92.615 |  |  |  |
| 12 | .299 | 1.757 | 94.371 |  |  |  |
| 13 | .254 | 1.493 | 95.864 |  |  |  |
| 14 | .236 | 1.386 | 97.250 |  |  |  |
| 15 | .198 | 1.166 | 98.416 |  |  |  |
| 16 | .150 | .883 | 99.299 |  |  |  |
| 17 | .119 | .701 | 100.000 |  |  |  |
| ***Note:***  *Extraction Method:* Principal Axis Factoring. | | | | | | |

**Table C: Common Latent Factor Analysis for Common Method Variance**

| **Relationship** | **With CLF** | **Without CLF** | **Change**  **(Without CLF – With CLF)** |
| --- | --- | --- | --- |
|  | **Estimate** | **Estimate** |  |
| EE4 🡨 EE | 0.615 | 0.737 | 0.122 |
| EE3 🡨 EE | 0.719 | 0.831 | 0.112 |
| CCB1 🡨 CCB | 0.57 | 0.652 | 0.082 |
| CCB2 🡨 CCB | 0.661 | 0.75 | 0.089 |
| CCB3 🡨 CCB | 0.586 | 0.726 | 0.14 |
| CCB5 🡨 CCB | 0.642 | 0.739 | 0.097 |
| PCS3 🡨 PCS | 0.635 | 0.812 | 0.177 |
| PCS2 🡨 PCS | 0.571 | 0.744 | 0.173 |
| PCS1 🡨 PCS | 0.628 | 0.749 | 0.121 |
| AS3 🡨 AAAS | 0.784 | 0.932 | 0.148 |
| AS4 🡨 AAAS | 0.746 | 0.888 | 0.142 |
| AS2 🡨 AAAS | 0.584 | 0.754 | 0.17 |
| AS9 🡨 PAAS | 0.719 | 0.842 | 0.123 |
| AS8 🡨 PAAS | 0.663 | 0.832 | 0.169 |
| EE1 🡨 EE | 0.767 | 0.86 | 0.093 |
| EE2 🡨 EE | 0.736 | 0.841 | 0.105 |
| AS7 🡨 PAAS | 0.663 | 0.826 | 0.163 |

**Table D: Model Comparison**

| **Measurement Models** | **χ2** | **Δχ2** | **CFI** | **IFI** |
| --- | --- | --- | --- | --- |
| **Five Factors *(Base model)*** | 293.623*** | - | 0.912 | 0.914 |
| **Four Factors - A *(Combined AAAS and PAAS into one factor)*** | 373.354*** | 79.731*** | 0.882 | 0.883 |
| **Four Factors - B *(Combined CCB and EE into one factor)*** | 457.20*** | 163.577*** | 0.844 | 0.846 |
| **Three Factors *(Combined CCB and EE into one factor and AAAS and PAAS into one factor)*** | 493.148*** | 199.525*** | 0.829 | 0.831 |
| **Two Factors *(Combined PCS, EE and CCB into one factor and AAAS and PAAS into one factor)*** | 688.359*** | 394.736*** | 0.742 | 0.744 |
| **Single Factor** | 913.498*** | 619.875*** | 0.641 | 0.643 |
| Note :  **** p <0.01* | | | | |

**Table E: Model Fit Indices of Five Factor Model**

| **Model Fit Indices** | **Recommended** | **Actual** | **Reference** |
| --- | --- | --- | --- |
| **Absolute Fit Indices** |  | |  |
| ***χ2*** | - | 293.623*** | - |
| ***CMIN/df*** | 1 - 3 | 2.93 | Hair et al. (2010) |
| **Incremental Fit Indices** |  | |  |
| **IFI** | > 0.90 | 0.914 |  |
| **CFI** | > 0.90 | 0.912 |  |
| **** p <0.01* | | | |

**Table F: Latent Constructs with Standardized Factor Loadings**

| **Constructs** | **Items** | **Standardized Factor Loadings** | **Cronbach’s Alpha** | **CR** | **AVE** | **ASV** |
| --- | --- | --- | --- | --- | --- | --- |
| ***Active-Aggressive Abusive Supervision*** | **AS1** | Dropped | 0.882 | 0.895 | 0.742 | 0.396 |
|  | **AS2** | 0.754 |  |  |  |  |
|  | **AS3** | 0.932 |  |  |  |  |
|  | **AS4** | 0.888 |  |  |  |  |
|  | **AS5** | Dropped |  |  |  |  |
| ***Passive-Aggressive Abusive Supervision*** | **AS6** | Dropped | 0.830 | 0.872 | 0.694 | 0.397 |
|  | **AS7** | 0.826 |  |  |  |  |
|  | **AS8** | 0.832 |  |  |  |  |
|  | **AS9** | 0.842 |  |  |  |  |
|  | **AS10** | Dropped |  |  |  |  |
| ***Perceived Coworker's Support*** | **PCS1** | 0.749 | 0.805 | 0.812 | 0.591 | 0.037 |
|  | **PCS2** | 0.744 |  |  |  |  |
|  | **PCS3** | 0.812 |  |  |  |  |
| ***Emotional Exhaustion*** | **EE1** | 0.86 | 0.902 | 0.890 | 0.670 | 0.337 |
|  | **EE2** | 0.841 |  |  |  |  |
|  | **EE3** | 0.831 |  |  |  |  |
|  | **EE4** | 0.737 |  |  |  |  |
|  | **EE5** | Dropped |  |  |  |  |
| ***Compulsory Citizenship Behavior*** | **CCB1** | 0.652 | 0.807 | 0.809 | 0.515 | 0.373 |
|  | **CCB2** | 0.75 |  |  |  |  |
|  | **CCB3** | 0.726 |  |  |  |  |
|  | **CCB4** | Dropped |  |  |  |  |
|  | **CCB5** | 0.739 |  |  |  |  |

**Table G: Research Paper Summaries**

| **No.** | **Independent Variable** | **Dependent Variable** | **Mediator** | **Moderator** | **Findings** | **Reference** |
| --- | --- | --- | --- | --- | --- | --- |
| 1 | Abusive Supervision | Emotional Exhaustion | - | Perceived Coworker’s Support; Susceptibility to emotional contagion | Significant positive relationship between AS and EE. PCS moderates the relationship | Wu et al., (2007) |
| 2 | Abusive Supervision | Emotional Exhaustion; Job Satisfaction; Job Tension | - | Accountability | Significant positive relationship between AS and EE. | Breaux et al., (2008) |
| 3 | Abusive Supervision | Emotional Exhaustion; Engagement | Challenge Demand Appraisals; Hindrance Demand Appraisals | - | Significant positive relationship between AS and EE. | Scheuer et al., (2016) |
| 4 | Citizenship Pressure; OCB; | Citizenship Fatigue | - | Perceived organizational Support; TMX | Significant Positive Relationship between CP and CF. | Bolino et al., (2015) |
| 5 | Abusive Supervision | Compulsory Citizenship Behavior | Psychological Safety | Chinese Traditionality | AS has significant positive relationship with CCB | Zhao et al., (2013) |
| 6 | Abusive Supervision | Contextual Performance | Emotional Exhaustion | Work Unit Structure | AS has significant negative relationship with contextual performance. Emotional Exhaustion mediates this relationship | Aryee et al., (2008) |
| 7 | Citizenship Pressure | Compulsory Citizenship Behavior | - | Job Autonomy | CP has positive relationship with CCB | Liu et al., (2017) |
| 8 | Abusive Supervision | Job Performance | - | Meaning of Work | AS has significant negative relationship with job performance | Harris et al., (2007) |
| 9 | Abusive Supervision | Turnover Intention | Emotional Exhaustion | Perceived Coworker’s Support | High CWS buffer negative impact from AS to EE | Pradhan & Jena, (2018) |
| 10 | Abusive Supervision | Contextual Performance | Emotional Exhaustion | Work Unit Structure | AS has significant negative relationship with contextual performance. Emotional Exhaustion mediates this relationship | Aryee et al., (2008) |
| 11 | Abusive Supervision | Turnover Intention | Emotional Exhaustion | Perceived Coworker’s Support | Moderated Mediation supported | Pradhan & Jena (2018) |
| 12 | Abusive Supervision | Counterproductive Work behavior | Emotional Exhaustion | Job Demands | Moderated Mediation Model supported | Akram et al., (2019) |
| 13 | Work-Family Conflict, Family-Work Conflict | Turnover Intention | - | Perceived Family Supportive Supervisor | Family supportive supervisor behaviors moderates the relationship of family-work conflict and Turnover intentions | Asghar et al., (2018) |
| 14 | Polychronicity | Job Performance, Turnover Intention | Work Engagement | Perceived Organizational Support | Polychronic employees with a high level of POS can handle multitasking and task switching to cope TI | Asghar et al., (2021) |
